# Laparoscopic Watson Fundoplication Is Effective and Durable in Children with Gastrooesophageal Reflux

**DOI:** 10.1155/2014/409727

**Published:** 2014-12-31

**Authors:** Matthew G. Dunckley, Kapil M. Rajwani, Anies A. Mahomed

**Affiliations:** Department of Paediatric Surgery, Royal Alexandra Children's Hospital, Brighton BN2 5BE, UK

## Abstract

Gastroesophageal reflux (GOR) affects 2–8% of children over 3 years of age and is associated with significant morbidity. The disorder is particularly critical in neurologically impaired children, who have a high risk of aspiration. Traditionally, the surgical antireflux procedure of choice has been Nissen's operation. However, this technique has a significant incidence of mechanical complications and has a reoperation rate of approximately 7%, leading to the development of alternative approaches. Watson's technique of partial anterior fundoplication has been shown to achieve long-lasting reflux control in adults with few mechanical complications, but there is limited data in the paediatric population. We present here short- and long-term outcomes of laparoscopic Watson fundoplication in a series of 76 children and infants, 34% of whom had a degree of neurological impairment including severe cerebral palsy and hypoxic brain injury. The overall complication rate was 27.6%, of which only 1 was classified as major. To date, we have not recorded any incidences of perforation and no revisions. In our experience, Watson's laparoscopic partial fundoplication can be performed with minimal complications and with durable results, not least in neurologically compromised children, making it a viable alternative to the Nissen procedure in paediatric surgery.

## 1. Introduction

Gastroesophageal reflux (GOR) affects a large number of infants and children. For the majority, the condition is self-limiting and most patients improve spontaneously over the first years of life. However, it persists in 2–8% of children aged 2–17 years [1]. Medical therapy is the first line treatment of choice, but surgery remains an option if this fails to control reflux adequately [2]. Indications for surgical treatment include failed medical therapy, the presence of reflux-related respiratory complications, or failure to thrive. In older children, in whom reflux is unlikely to resolve spontaneously, there is generally a reluctance to accept lifelong proton pump inhibitors (PPIs) and quality of life issues become more prominent. The recognition that the placement of gastrostomy tubes for feeding significantly increases the risk of GOR, together with evidence that reflux may play an important role in the occurrence of apnoeic or bradycardic episodes, sudden death, recurrent chest infections, and chronic reactive airway disease, has also resulted in a large increase in antireflux surgery [3].

Laparoscopic antireflux surgery is now firmly established as a useful tool in the management of GOR in children, as it is associated with less morbidity and a more rapid recovery, particularly in children with neurological impairment [4–6]. Several antireflux procedures have been described, but which of these techniques offer the best outcomes in children has been open to debate. Nissen's fundoplication was established prior to the advent of laparoscopic surgery, so this technique was the first to be adapted for laparoscopic surgery and, consequently, has been the most widely used by laparoscopic surgeons [7–9]. Nevertheless, a high incidence of mechanical side effects has been reported, such as dysphagia, an inability to belch or vomit, and gas bloating, all of which are likely due to the establishment of a supercompetent gastrooesophageal sphincter by the 360-degree wrap [9]. As a result, alternative fundoplication techniques have been developed by Boix-Ochoa, Toupet, and others. However, which of these techniques offer the best outcomes in children is still under debate [10]. One of the modifications of the laparoscopic Nissen technique, using a partial anterior wrap, was described by Watson in 1991 [11]. This method is associated with reduced mechanical side effects in adults [12], but its efficacy in children has not yet been firmly established.

The aim of this study was to evaluate the short- and long-term outcomes of laparoscopic Watson fundoplication in children and infants in terms of durability and the incidence of mechanical complications, especially in those children with neurological impairment.

## 2. Materials and Methods 

This was a prospective observational study of 76 children and infants who had laparoscopic Watson fundoplication performed by a single surgeon at the Royal Aberdeen Children's Hospital and the Royal Alexandra Children's Hospital in Brighton between 1995 and 2014. Demographic data, investigations, operative technique, and all reported complications were collected and analysed using Microsoft Excel. All patients were investigated with barium swallow and 89% had preoperative endoscopy (see Table 1). Where necessary, the degree of reflux was clarified by oesophageal pH and manometry studies, where acid reflux was defined by a Demeester score > 14.72 [13].

All patients received a single dose of 25 mg/kg co-amoxiclav (or 30 mg/kg cefotaxime if allergic to penicillin). Laparoscopy utilised a four-port technique with an additional epigastric stab incision to accommodate a Nathanson liver retractor. All umbilical camera ports were inserted using the Hasson technique. A 10 mm port was used in older children and a 5 mm port in small children and infants, to accommodate either a 0-degree or 30-degree laparoscope. Once pneumoperitoneum was established, two instrument ports were placed either side of the umbilicus for triangulation using either 5 mm or 3.5 mm ports for older or younger children, respectively. A further 5 mm or 3.5 mm port was placed near the left anterior axillary line to retract the gastrooesophageal junction.

In children, spatial restrictions necessitate accessing the hiatus by dissection of the lesser omentum in the initial instance. Adhesions between the oesophagus and right crus were then released and the inferior oesophagus mobilised by extended dissection into the posterior mediastinum. The phreno-oesophageal ligament was divided anteriorly before the oesophagus was brought down into the peritoneum, taking care to avoid damage to the anterior vagus nerve. Next, the fundus was drawn over to the patient's right with division of phreno-fundal adhesions to release the left crus. Posterior dissection proceeded at the cardiooesophageal junction, avoiding the posterior vagus nerve. A nylon tape was then used as a sling and held via the left anterior axillary port. Once 4-5 cm of tension-free oesophagus was brought down into the peritoneum, the crural repair was performed with two or three 2/0 Ethibond sutures. The angle of His was recreated by suturing the fundus to the left anterior oesophageal hiatal rim. Further fundal tissue was then rolled over the anterior oesophagus and sutured to the mid portion and right angle of the anterior hiatal rim to create a 180-degree anterior wrap. Finally, three sutures were passed from the rolled-over fundus through the right oesophageal wall to the right crural repair. Half circle needles were used in younger children and infants and “ski needles” in older children. Postoperatively, patients were allowed clear fluids via oral or gastrostomy routes on the same day as surgery and allowed feeds the following day. All families received dietary advice prior to discharge.

Follow up included standard post-operative outpatient appointments at 6 weeks, 3 months and, where possible, at least one annual review. Specific questions were asked to establish the incidence of adverse mechanical symptoms. Treatment failure was defined as the continued or renewed dependence on PPIs, histamine H2 receptor antagonists, or regular antacids for reflux symptoms as well as wrap failure with reoperation.

## 3. Results

76 patients (50 male, 26 female) were included. The mean age at operation was 8.2 years (range 0.2–18 years). 42 (55.2%) patients suffered from significant comorbidity. Thirty-three (34.2%) were neurologically impaired, one suffered from cystic fibrosis, and 6 were born with oesophageal atresia +/− tracheooesophageal fistula. Fifteen patients (19.7%) had previously undergone abdominal or thoracic surgery or percutaneous endoscopic gastrostomy (PEG) placement. One child had previously undergone Nissen fundoplication by a nonpaediatric specialist and represented with recurrent symptoms and a further patient underwent a failed Boix-Ochoa fundoplication by a paediatric surgeon. Contrast studies in these patients confirmed failure of the wrap and recurrence of hiatus hernia. All patients were investigated preoperatively by contrast radiology. The majority of children (*n* = 68; 89.4%) also underwent upper gastrointestinal endoscopy and 44 (57.9%) patients had pH studies. A summary of outcomes is presented in Table 2.

### 3.1. Early (<30 Days) and Short-Term (1–12 Months) Outcomes

Over 72% of patients had an uncomplicated laparoscopic Watson fundoplication and recovery. Median postoperative stay was 2 days (mean 3 days; range 1–19 days). There were no major intraoperative complications and no conversions to open surgery. Of the 76 patients undergoing surgery, 21 (27.6%) had a gastrostomy placed at the same time. One of these neurologically compromised patients with delayed gastric emptying experienced a gastrostomy leak. Immediate complications included 4 minor liver injuries (5.25%), 6 minor bleeds (7.9%), and 1 bleed requiring a postoperative transfusion. In the short- to medium-term, one patient developed a wound infection and one had an early port site hernia. Postoperatively, dysphagia was reported in 9 cases (11.8%), 5 of whom had confirmation of relative narrowing at the cardiooesophageal junction at the level of the wrap on barium swallow.

### 3.2. Long-Term (>12 Months) Outcomes

The median duration of follow-up was 30 months (range 0–43 months). 22 patients were followed up for over 1 year. One child developed late dysphagia and was found to have a recurrent stricture proximal to the wrap on barium swallow, requiring endoscopic balloon dilatation. This patient had a preoperative diagnosis of achalasia with oesophageal stricturing. Reflux symptoms (arching, postfeed cough, or vomiting) were reported in 14 cases (18.4%), but only 2 had acid reflux confirmed by pH studies. Two cases developed a small hiatus hernia managed medically, 3 cases suffered recurrent retching again managed medically, and a high risk older cerebral palsy patient with respiratory compromise had a gastrojejunostomy placed for breakthrough chest aspiration after spinal surgery.

A patient who had undergone a tracheooesophageal fistula (TOF) repair as a neonate and who had been listed for fundoplication due to frequent apnoeas, thought to be reflux-related, continued to experience apnoeas postoperatively, but at a reduced frequency.

## 4. Discussion

Laparoscopic fundoplication for the management of GOR in infants and children has become one of the most frequently performed operations in paediatric surgery, as its effectiveness and safety have been well established [14]. Nissen's technique remains the most commonly performed antireflux operation in children. In adult practice, laparoscopic anterior fundoplication appears to be associated fewer adverse events, especially mechanical complications, than Nissen fundoplication [15–17]. The advantages should, in theory, apply to paediatric practice as well. To date, however, only a limited number of patients series with children or infants undergoing anterior fundoplication have been published [14, 18–21].

The evaluation of antireflux surgery postoperatively is notoriously difficult. Comparison of complications and treatment failures are hampered by the lack of precise definitions of symptoms, such as “gas bloat” and “early dysphagia.” Even “treatment failure” is variously defined as wrap breakdown on contrast swallow, recurrence of symptoms, persistent or recurrent reflux on pH studies, and reinstitution of medical therapy or reoperation. In our unselected consecutive series of patients, none experienced wrap failure. Despite the Watson fundoplication being a strictly anterior wrap, postoperative dysphagia is seen in approximately 12% of cases, but it is nearly always transient and rarely requires oesophageal dilatation. Although 18.4% reported ongoing or recurrent reflux, this figure appears to be satisfactory relative to other reports [12–17]. Furthermore, the incidence of early dysphagia in our series falls between that reported by other authors for Nissen fundoplication [10, 17], although there are marked differences in study design.

Over a third of the children in our series had neurological comorbidities. The outcome of antireflux surgery in children with neurological impairment is known to be inferior to that of neurologically intact children [4–6]. Many of these children are accepted for surgical intervention on the basis of vomiting or failure to thrive, which may be due to causes other than reflux. More thorough preoperative investigations and distinction of centrally induced retching from true reflux might have altered the decision to proceed to surgery in some of our patients. Indeed, in adults, most surgeons would now be reluctant to perform antireflux surgery without convincing evidence of high volume reflux on endoscopy, contrast swallow, and/or pH-manometry studies [22]. Despite the fact that reliable pH studies are difficult to perform in children, especially in those unable to self-report symptom events, consideration should be given to obtaining such objective evidence prior to surgery, especially if the indications are nonspecific. Neurologically compromised patient are principally gastrostomy fed so rarely experience dysphagia.

## 5. Conclusion

This study confirms that the technique of laparoscopic Watson fundoplication is safe in infants and children, including those with significant neurological disability. Adverse events, although relatively common, were generally very mild and tended to be transient. Long-term outcomes were good in over 80% of cases.

## Figures and Tables

**Table 1:** Investigations of patient symptoms (*n* = 76; percentages in parentheses).

	Preoperative	Postoperative
Endoscopy	68 (89.4)	2 (2.6)
pH study	44 (58)	2 (2.6)
Barium swallow	76 (100)	5 (6.6)

**Table 2:** All postoperative complications following laparoscopic Watson fundoplication (*n* = 76; percentages in parentheses).

	Early (<30 days) (*n* = 76)	Medium-term (1–12 months) (*n* = 68)	Long-term (>12 months) (*n* = 22)
Dysphagia^*^	9 (11.8)	5 (7.3)	0
Gas bloat	6 (7.9)	1 (1.5)	0
Delayed emptying	2 (2.6)	0	0
Transfusion	1 (1.3)	0	0
Apnoeas^**^	1 (1.3)	1 (1.5)	0
Acid reflux	0	2 (2.9)	0
Wound infection	2 (2.6)	0	1 (4.5)
Hiatus hernia	0	0	2 (9)

Total	21 (27.6)	9 (13.2)	2 (9)

^*^Most were mild dysphagia. Only 1 patient had a demonstrable abnormality on barium study which resolved following dilatation. ^**^This patient suffered apnoeas preoperatively. The frequency of apnoeic episodes reduced postoperatively.
